# (*E*)-*N*-(1-Benzothio­phen-3-yl­methyl­idene)-2,6-dimethyl­aniline

**DOI:** 10.1107/S1600536812004151

**Published:** 2012-02-04

**Authors:** Nermin Kahveci Yağcı, Necmi Dege, Sümeyye Gümüş, Erbil Ağar, Mustafa Serkan Soylu

**Affiliations:** a Kırıkkale University, Faculty of Arts and Sciences, Physics Department, 71450 Kırıkkale, Turkey; b Ondokuz Mayıs University, Arts and Sciences Faculty, Department of Physics, 55139 Samsun, Turkey; c Ondokuz Mayıs University, Arts and Sciences Faculty, Department of Chemistry, 55139 Samsun, Turkey; d Giresun University, Faculty of Arts and Sciences, Department of Physics, 28100 Giresun, Turkey

## Abstract

In the title compound, C_17_H_15_NS, the benzothio­phene residue and the substituted benzene ring are oriented at a dihedral angle of 61.99 (7)°. An inter­molecular C—H⋯π inter­action contributes to the stability of the crystal structure.

## Related literature

For the biological properties of Schiff bases, see: Barton & Ollis (1979 ▶); Layer (1963 ▶); Ingold (1969 ▶). For industrial applications of Schiff bases, see: Taggi *et al.* (2002 ▶). For chemical properties of Schiff bases, see: Aydoğan *et al.* (2001 ▶); Tanak *et al.* (2010 ▶); Ingold (1969 ▶). For related structures, see: Ağar *et al.* (2010 ▶); Ceylan *et al.* (2011 ▶); Dege, Şekerci *et al.* (2006 ▶); Demirtaş *et al.* (2009 ▶); Dege, Içbudak & Adıyaman (2006 ▶, 2007 ▶); Genç *et al.* (2004 ▶); İnaç *et al.* (2012 ▶); Tecer *et al.* (2010 ▶). For the structural properties benzothiophene derivatives, see: Alarcon *et al.* (1999 ▶); Cohen *et al.* (1964 ▶); Hadjoudis *et al.* (1987 ▶); Inamoto *et al.* (2008 ▶); Köysal *et al.* (2007 ▶); Karabıyık *et al.* (2008 ▶); Kobayashi *et al.* (2009 ▶); Mlochowski & Potaczek (2009 ▶); Novopoltseva (1995 ▶); Tanak *et al.* (2010 ▶); Xu *et al.* (1994 ▶); Zhang *et al.* (2001 ▶).
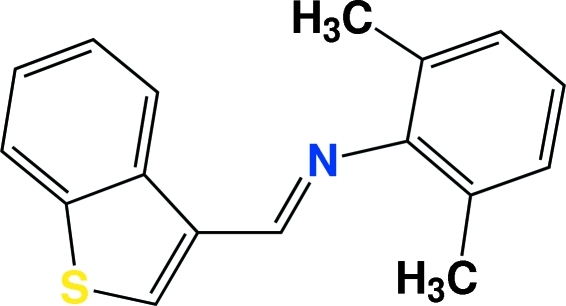


## Experimental

### 

#### Crystal data

C_17_H_15_NS
*M* *_r_* = 265.36
Monoclinic, 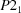

*a* = 8.0446 (10) Å
*b* = 8.8031 (9) Å
*c* = 10.2809 (10) Å
β = 98.989 (11)°
*V* = 719.12 (14) Å^3^
*Z* = 2
Mo *K*α radiation
μ = 0.21 mm^−1^
*T* = 296 K
0.17 × 0.15 × 0.12 mm

#### Data collection

Oxford Diffraction SuperNova (single source at offset) Eos diffractometer
Absorption correction: multi-scan (*CrysAlis PRO*; Oxford Diffraction, 2007 ▶) *T*_min_ = 0.965, *T*_max_ = 0.975
2788 measured reflections
2024 independent reflections
1722 reflections with *I* > 2σ(*I*)
*R*_int_ = 0.021

#### Refinement

*R*[*F* ^2^ > 2σ(*F* ^2^)] = 0.040
*wR*(*F* ^2^) = 0.101
*S* = 1.05
2024 reflections
174 parameters
1 restraint
H-atom parameters constrained
Δρ_max_ = 0.15 e Å^−3^
Δρ_min_ = −0.23 e Å^−3^
Absolute structure: Flack (1983 ▶), 497 Friedel pairs
Flack parameter: 0.10 (11)

### 

Data collection: *CrysAlis PRO* (Oxford Diffraction, 2007 ▶); cell refinement: *CrysAlis PRO*; data reduction: *CrysAlis PRO*; program(s) used to solve structure: *SHELXS97* (Sheldrick, 2008 ▶); program(s) used to refine structure: *SHELXL97* (Sheldrick, 2008 ▶); molecular graphics: *OLEX2* (Dolomanov *et al.*, 2009 ▶) and *ORTEP-3 for Windows* (Farrugia, 1997 ▶); software used to prepare material for publication: *OLEX2*, *WinGX* (Farrugia, 1999 ▶) and *PLATON* (Spek, 2009 ▶).

## Supplementary Material

Crystal structure: contains datablock(s) I, global. DOI: 10.1107/S1600536812004151/bt5806sup1.cif

Structure factors: contains datablock(s) I. DOI: 10.1107/S1600536812004151/bt5806Isup2.hkl

Supplementary material file. DOI: 10.1107/S1600536812004151/bt5806Isup3.cml

Additional supplementary materials:  crystallographic information; 3D view; checkCIF report

## Figures and Tables

**Table 1:** Hydrogen-bond geometry (Å, °) *Cg*2 is the centroid of the C1–C6 ring.

*D*—H⋯*A*	*D*—H	H⋯*A*	*D*⋯*A*	*D*—H⋯*A*
C9—H9⋯*Cg*2^i^	0.93	2.95	3.872 (4)	171

Symmetry code: (i) 
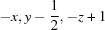
.

**Fig. 1.**
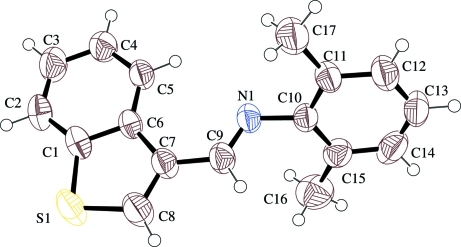
The molecular structure of the title compound, showing the atom-numbering scheme and 50% probability displacement ellipsoids.

## supplementary crystallographic information

### Comment

Benzothiophene derivatives are undoubtedly one of the most important classes of heterocycles, because some of molecules having the benzothiophene skeleton have been reported to axhibit wide variety of biological activities (Kobayashi *et al.*, 2009). Therefore, we (İnaç *et al.* 2012) and others (İnamoto *et al.*, 2008; Mlochowski & Potaczek, 2009) have recently reported efficient methods for the synthesis of benzothiophenes. However, there are only a few reports on the synthesis of 1-(benzo[*b*]thiophen-3-yl)-*N*-methylmethanamines, which may also be of potential biological importance.

Schiff bases, *i.e.*, compounds having a double C=N bond, are used as starting materials in the synthesis of important drugs, such as antibiotics and antiallergic, antiphlogistic, and antitumor substances (Barton & Ollis, 1979; Layer, 1963; Ingold 1969). On the industrial scale, they have a wide range of applications, such as dyes and pigments (Taggi *et al.*, 2002). They are also used as components of rubber compounds (Novopoltseva, 1995). Schiff base compounds display interesting photochromic and thermochromic features in the solid state and can be classified in terms of these properties (Cohen *et al.*, 1964). Photo- and thermochromism arise *via* H-atom transfer from the hydroxy O atom to the imine N atom (Hadjoudis *et al.*, 1987;Xu *et al.*, 1994). Such proton-exchanging materials can be utilized for the design of various molecular electronic devices (Alarcon *et al.*, 1999). In general, Schiff bases display two possible tautomeric forms, the phenol-imine (OH) and the keto-amine (NH) forms. Depending on the tautomers, two types of intramolecular hydrogen bonds are observed in Schiff bases: O—H···N in phenol-imine (Köysal *et al.*, 2007) and N—H···O in keto-amine tautomers. By means of increasing development of computational chemistry in the past decade, the research of theoretical modeling of drug design, functional material design, *etc*., has become more mature than ever. Many important chemical and physical properties of biological and chemical systems can be predicted from the first principles by various computational techniques (Zhang *et al.*, 2001). Schiff bases have also been employed as ligands for the complexation of metal ions (Aydoğan *et al.*, 2001).

The molecular structure is not planar (Fig.1); the dihedral angle between the C10—C17 benzene and the C1—C8/S1 benzothiophene ring is 61.99 (7)°. The dihedral angle between the methylenemethanamine and bezothiophene group is 3.22 (50)°. The length of the C9=N1 double bond is 1.260 (3) Å, slightly shorter than standard 1.28 Å value of a C=N double bond and consistent with related structures (Ağar *et al.*, 2010; Ceylan *et al.* 2011; Dege, Şekerci *et al.*, 2006; Genç *et al.* 2004; İnaç *et al.*, 2012; Tanak *et al.*, 2010; Tecer *et al.*, 2010).

The C1—S1 and C8—S1 bond distances are 1.736 (3) Å and 1.717 (3) Å, respectively. The C—S bond distances are compatible with the literature (Dege, Içbudak & Adıyaman, 2006, 2007; Demirtaş *et al.*, 2009).

The crystal structure is stabilized by an intermolecular C—H···π stacking interaction (C9—H9···*Cg*2^i^ = 2.95 Å) [symmetry code (i): -*x*, -1/2 + *y*, 1 - *z*; *Cg*2 is the centroids of ring C1—C6].

### Experimental

The compound (*E*)-1-(1-benzothiophen-3-yl)-*N*-(2,6-dimethylphenyl)methanimine was prepared by reflux a mixture of a solution containing 1-benzothiophene-3-carbaldehyde (0.026 g 0.16 mmol) in 20 ml e thanol and a solution containing 2,6-Dimethylaniline (0.0192 g 0.16 mmol) in 20 ml e thanol. The reaction mixture was stirred for 1 hunder reflux. The crystals of 2(*E*)-1-(1-benzothiophen-3-yl)-*N*-(2,6-dimethylphenyl)methanimine suitable for X-ray analysis were obtained from ethylalcohol by slow evaporation (yield % 58; m.p 75–77°C).

### Refinement

All hydrogen atoms were positioned geometrically [C—H=0.930 and 0.960] and treated as riding with *U*_iso_(H)=1.2*U*_eq_(C) or 1.5*U*_eq_(C_methyl_).

### Crystal data

**Table:**

C_17_H_15_NS	*F*(000) = 280
*M**_r_* = 265.36	*D*_x_ = 1.225 Mg m^−^^3^
Monoclinic, *P*2_1_	Mo *K*α radiation, λ = 0.71073 Å
Hall symbol: P 2yb	Cell parameters from 1387 reflections
*a* = 8.0446 (10) Å	θ = 3.5–27.3°
*b* = 8.8031 (9) Å	µ = 0.21 mm^−^^1^
*c* = 10.2809 (10) Å	*T* = 296 K
β = 98.989 (11)°	Prism, brown
*V* = 719.12 (14) Å^3^	0.17 × 0.15 × 0.12 mm
*Z* = 2	

### Data collection

**Table:**

Oxford Diffraction SuperNova (single source at offset) Eos diffractometer	2024 independent reflections
Radiation source: SuperNova (Mo) X-ray Source	1722 reflections with *I* > 2σ(*I*)
Mirror monochromator	*R*_int_ = 0.021
Detector resolution: 16.0454 pixels mm^-1^	θ_max_ = 27.4°, θ_min_ = 3.5°
ω scans	*h* = −10→5
Absorption correction: multi-scan (*CrysAlis PRO*; Oxford Diffraction, 2007)	*k* = −5→11
*T*_min_ = 0.965, *T*_max_ = 0.975	*l* = −10→13
2788 measured reflections	

### Refinement

**Table:**

Refinement on *F*^2^	Secondary atom site location: difference Fourier map
Least-squares matrix: full	Hydrogen site location: inferred from neighbouring sites
*R*[*F*^2^ > 2σ(*F*^2^)] = 0.040	H-atom parameters constrained
*wR*(*F*^2^) = 0.101	*w* = 1/[σ^2^(*F*_o_^2^) + (0.0397*P*)^2^ + 0.1075*P*] where *P* = (*F*_o_^2^ + 2*F*_c_^2^)/3
*S* = 1.05	(Δ/σ)_max_ < 0.001
2024 reflections	Δρ_max_ = 0.15 e Å^−^^3^
174 parameters	Δρ_min_ = −0.23 e Å^−^^3^
1 restraint	Absolute structure: Flack (1983), 497 Friedel pairs
Primary atom site location: structure-invariant direct methods	Flack parameter: 0.10 (11)

### Special details

**Table:**

Geometry. All e.s.d.'s (except the e.s.d. in the dihedral angle between two l.s. planes) are estimated using the full covariance matrix. The cell e.s.d.'s are taken into account individually in the estimation of e.s.d.'s in distances, angles and torsion angles; correlations between e.s.d.'s in cell parameters are only used when they are defined by crystal symmetry. An approximate (isotropic) treatment of cell e.s.d.'s is used for estimating e.s.d.'s involving l.s. planes.
Refinement. Refinement of *F*^2^ against ALL reflections. The weighted *R*-factor *wR* and goodness of fit *S* are based on *F*^2^, conventional *R*-factors *R* are based on *F*, with *F* set to zero for negative *F*^2^. The threshold expression of *F*^2^ > σ(*F*^2^) is used only for calculating *R*-factors(gt) *etc*. and is not relevant to the choice of reflections for refinement. *R*-factors based on *F*^2^ are statistically about twice as large as those based on *F*, and *R*- factors based on ALL data will be even larger.

### Fractional atomic coordinates and isotropic or equivalent isotropic displacement parameters (Å^2^)

**Table:**

	*x*	*y*	*z*	*U*_iso_*/*U*_eq_	
S1	0.82893 (13)	0.27137 (12)	0.24201 (7)	0.0801 (3)	
N1	0.9883 (3)	0.2484 (3)	0.7306 (2)	0.0511 (6)	
C10	1.0962 (3)	0.1995 (3)	0.8467 (2)	0.0491 (7)	
C7	0.9176 (4)	0.2495 (4)	0.4948 (2)	0.0565 (7)	
C6	0.7680 (3)	0.3424 (3)	0.4760 (2)	0.0453 (6)	
C11	1.0246 (4)	0.1127 (4)	0.9370 (3)	0.0567 (8)	
C5	0.6838 (4)	0.4137 (4)	0.5683 (3)	0.0560 (8)	
H5	0.7222	0.4028	0.6579	0.067*	
C2	0.5615 (4)	0.4471 (4)	0.2995 (3)	0.0659 (9)	
H2	0.5210	0.4585	0.2103	0.079*	
C13	1.2877 (5)	0.1134 (5)	1.0815 (3)	0.0766 (11)	
H13	1.3528	0.0846	1.1605	0.092*	
C15	1.2650 (4)	0.2449 (4)	0.8730 (3)	0.0590 (8)	
C1	0.7043 (4)	0.3622 (3)	0.3416 (3)	0.0551 (8)	
C9	1.0162 (4)	0.2036 (4)	0.6197 (3)	0.0575 (8)	
H9	1.1054	0.1370	0.6173	0.069*	
C14	1.3571 (4)	0.2000 (5)	0.9925 (4)	0.0784 (12)	
H14	1.4692	0.2294	1.0129	0.094*	
C3	0.4813 (5)	0.5139 (5)	0.3930 (3)	0.0772 (11)	
H3	0.3835	0.5696	0.3671	0.093*	
C12	1.1233 (4)	0.0697 (4)	1.0539 (3)	0.0695 (9)	
H12	1.0771	0.0105	1.1141	0.083*	
C4	0.5443 (4)	0.4995 (5)	0.5254 (3)	0.0698 (10)	
H4	0.4904	0.5494	0.5869	0.084*	
C8	0.9607 (5)	0.2058 (4)	0.3779 (3)	0.0772 (11)	
H8	1.0539	0.1452	0.3720	0.093*	
C17	0.8439 (4)	0.0649 (6)	0.9089 (4)	0.0854 (12)	
H17A	0.7741	0.1528	0.8880	0.128*	
H17B	0.8135	0.0155	0.9850	0.128*	
H17C	0.8282	−0.0040	0.8357	0.128*	
C16	1.3420 (5)	0.3460 (6)	0.7799 (4)	0.0923 (12)	
H16A	1.3583	0.2888	0.7033	0.138*	
H16C	1.4485	0.3834	0.8231	0.138*	
H16B	1.2681	0.4300	0.7538	0.138*	

### Atomic displacement parameters (Å^2^)

**Table:**

	*U*^11^	*U*^22^	*U*^33^	*U*^12^	*U*^13^	*U*^23^
S1	0.1139 (8)	0.0853 (7)	0.0394 (4)	0.0236 (6)	0.0066 (4)	−0.0016 (5)
N1	0.0541 (12)	0.0557 (15)	0.0416 (11)	0.0048 (12)	0.0016 (10)	0.0060 (12)
C10	0.0502 (14)	0.0525 (17)	0.0424 (14)	0.0067 (14)	0.0003 (12)	−0.0035 (13)
C7	0.0698 (18)	0.0563 (19)	0.0427 (13)	0.0091 (16)	0.0063 (13)	0.0045 (15)
C6	0.0565 (15)	0.0399 (15)	0.0375 (13)	−0.0026 (13)	0.0012 (11)	0.0045 (12)
C11	0.0616 (17)	0.065 (2)	0.0417 (15)	0.0004 (16)	0.0021 (13)	0.0005 (15)
C5	0.0612 (18)	0.062 (2)	0.0426 (15)	0.0031 (16)	0.0024 (13)	0.0041 (15)
C2	0.078 (2)	0.065 (2)	0.0483 (16)	0.0040 (18)	−0.0110 (16)	0.0069 (16)
C13	0.073 (2)	0.099 (3)	0.0517 (18)	0.025 (2)	−0.0101 (17)	0.003 (2)
C15	0.0512 (16)	0.064 (2)	0.0607 (16)	−0.0009 (16)	0.0049 (14)	−0.0074 (18)
C1	0.075 (2)	0.0468 (17)	0.0405 (15)	−0.0068 (16)	0.0007 (14)	0.0026 (13)
C9	0.0655 (18)	0.058 (2)	0.0466 (15)	0.0141 (16)	0.0024 (14)	0.0058 (14)
C14	0.0481 (16)	0.100 (3)	0.082 (2)	0.0119 (19)	−0.0083 (17)	−0.025 (2)
C3	0.075 (2)	0.085 (3)	0.066 (2)	0.020 (2)	−0.0052 (18)	0.008 (2)
C12	0.083 (2)	0.074 (2)	0.0495 (18)	0.015 (2)	0.0025 (17)	0.0097 (18)
C4	0.069 (2)	0.081 (3)	0.058 (2)	0.0190 (19)	0.0062 (17)	0.0024 (18)
C8	0.098 (3)	0.081 (3)	0.0517 (18)	0.028 (2)	0.0096 (17)	0.0013 (18)
C17	0.072 (2)	0.114 (3)	0.068 (2)	−0.025 (2)	0.0046 (19)	0.016 (2)
C16	0.077 (2)	0.099 (3)	0.102 (3)	−0.019 (2)	0.020 (2)	−0.005 (2)

### Geometric parameters (Å, º)

**Table:**

S1—C8	1.717 (3)	C13—C12	1.363 (5)
S1—C1	1.736 (3)	C13—C14	1.375 (5)
N1—C9	1.260 (3)	C13—H13	0.9300
N1—C10	1.428 (3)	C15—C14	1.390 (4)
C10—C11	1.395 (4)	C15—C16	1.509 (5)
C10—C15	1.400 (4)	C9—H9	0.9300
C7—C8	1.357 (4)	C14—H14	0.9300
C7—C6	1.442 (4)	C3—C4	1.382 (5)
C7—C9	1.456 (4)	C3—H3	0.9300
C6—C5	1.398 (4)	C12—H12	0.9300
C6—C1	1.407 (3)	C4—H4	0.9300
C11—C12	1.386 (4)	C8—H8	0.9300
C11—C17	1.497 (4)	C17—H17A	0.9600
C5—C4	1.367 (4)	C17—H17B	0.9600
C5—H5	0.9300	C17—H17C	0.9600
C2—C3	1.371 (5)	C16—H16A	0.9600
C2—C1	1.382 (4)	C16—H16C	0.9600
C2—H2	0.9300	C16—H16B	0.9600
			
C8—S1—C1	90.84 (15)	N1—C9—H9	117.9
C9—N1—C10	119.5 (2)	C7—C9—H9	117.9
C11—C10—C15	121.1 (2)	C13—C14—C15	122.0 (3)
C11—C10—N1	117.4 (2)	C13—C14—H14	119.0
C15—C10—N1	121.3 (3)	C15—C14—H14	119.0
C8—C7—C6	111.4 (2)	C2—C3—C4	120.7 (3)
C8—C7—C9	121.5 (3)	C2—C3—H3	119.6
C6—C7—C9	127.1 (2)	C4—C3—H3	119.6
C5—C6—C1	118.0 (3)	C13—C12—C11	120.6 (3)
C5—C6—C7	130.2 (2)	C13—C12—H12	119.7
C1—C6—C7	111.7 (2)	C11—C12—H12	119.7
C12—C11—C10	119.1 (3)	C5—C4—C3	121.6 (3)
C12—C11—C17	119.9 (3)	C5—C4—H4	119.2
C10—C11—C17	121.0 (3)	C3—C4—H4	119.2
C4—C5—C6	119.3 (3)	C7—C8—S1	114.5 (3)
C4—C5—H5	120.3	C7—C8—H8	122.7
C6—C5—H5	120.3	S1—C8—H8	122.7
C3—C2—C1	118.2 (3)	C11—C17—H17A	109.5
C3—C2—H2	120.9	C11—C17—H17B	109.5
C1—C2—H2	120.9	H17A—C17—H17B	109.5
C12—C13—C14	120.0 (3)	C11—C17—H17C	109.5
C12—C13—H13	120.0	H17A—C17—H17C	109.5
C14—C13—H13	120.0	H17B—C17—H17C	109.5
C14—C15—C10	117.2 (3)	C15—C16—H16A	109.5
C14—C15—C16	120.9 (3)	C15—C16—H16C	109.5
C10—C15—C16	121.8 (3)	H16A—C16—H16C	109.5
C2—C1—C6	122.1 (3)	C15—C16—H16B	109.5
C2—C1—S1	126.4 (2)	H16A—C16—H16B	109.5
C6—C1—S1	111.5 (2)	H16C—C16—H16B	109.5
N1—C9—C7	124.2 (3)		
			
C9—N1—C10—C11	−117.4 (3)	C5—C6—C1—S1	177.9 (2)
C9—N1—C10—C15	66.6 (4)	C7—C6—C1—S1	−1.2 (3)
C8—C7—C6—C5	−178.2 (3)	C8—S1—C1—C2	179.8 (3)
C9—C7—C6—C5	1.9 (6)	C8—S1—C1—C6	1.0 (2)
C8—C7—C6—C1	0.7 (4)	C10—N1—C9—C7	−178.1 (3)
C9—C7—C6—C1	−179.2 (3)	C8—C7—C9—N1	175.4 (4)
C15—C10—C11—C12	−0.7 (5)	C6—C7—C9—N1	−4.7 (5)
N1—C10—C11—C12	−176.8 (3)	C12—C13—C14—C15	−0.4 (6)
C15—C10—C11—C17	179.7 (3)	C10—C15—C14—C13	0.6 (5)
N1—C10—C11—C17	3.6 (5)	C16—C15—C14—C13	177.2 (4)
C1—C6—C5—C4	−0.2 (5)	C1—C2—C3—C4	1.5 (6)
C7—C6—C5—C4	178.7 (3)	C14—C13—C12—C11	−0.5 (6)
C11—C10—C15—C14	−0.1 (5)	C10—C11—C12—C13	1.0 (5)
N1—C10—C15—C14	175.8 (3)	C17—C11—C12—C13	−179.4 (4)
C11—C10—C15—C16	−176.6 (3)	C6—C5—C4—C3	2.0 (6)
N1—C10—C15—C16	−0.7 (5)	C2—C3—C4—C5	−2.7 (6)
C3—C2—C1—C6	0.3 (5)	C6—C7—C8—S1	0.1 (4)
C3—C2—C1—S1	−178.4 (3)	C9—C7—C8—S1	180.0 (3)
C5—C6—C1—C2	−1.0 (4)	C1—S1—C8—C7	−0.7 (3)
C7—C6—C1—C2	180.0 (3)		

### Hydrogen-bond geometry (Å, º)

Cg2 is the centroid of the (C1-C6) ring.

**Table:**

*D*—H···*A*	*D*—H	H···*A*	*D*···*A*	*D*—H···*A*
C9—H9···*Cg*2^i^	0.93	2.95	3.872 (4)	171

Symmetry code: (i) −*x*, *y*−1/2, −*z*+1.
